# Study on the Impact Resistance of Bionic Layered Composite of TiC-TiB_2_/Al from Al-Ti-B_4_C System

**DOI:** 10.3390/ma9080708

**Published:** 2016-08-20

**Authors:** Qian Zhao, Yunhong Liang, Zhihui Zhang, Xiujuan Li, Luquan Ren

**Affiliations:** 1 The Key Laboratory of Bionic Engineering, Ministry of Education, Jilin University, Changchun 130025, China; zrzhaoqian@163.com (Q.Z.); zhzh@jlu.edu.cn (Z.Z.); xiujuanli@jlu.edu.cn (X.L.); lqren@jlu.edu.cn (L.R.); 2 School of Mechanical, Aerospace and Civil Engineering, University of Manchester, Manchester M13 9PL, UK; 3 State Key Laboratory of Automotive Simulation and Control, Jilin University, Changchun 130025, China

**Keywords:** shell, bionic design, layered composite, mechanical property

## Abstract

Mechanical property and impact resistance mechanism of bionic layered composite was investigated. Due to light weight and high strength property, *white clam* shell was chosen as bionic model for design of bionic layered composite. The intercoupling model between hard layer and soft layer was identical to the layered microstructure and hardness tendency of the *white clam* shell, which connected the bionic design and fabrication. TiC-TiB_2_ reinforced Al matrix composites fabricated from Al-Ti-B_4_C system with 40 wt. %, 50 wt. % and 30 wt. % Al contents were treated as an outer layer, middle layer and inner layer in hard layers. Pure Al matrix was regarded as a soft layer. Compared with traditional homogenous Al-Ti-B_4_C composite, bionic layered composite exhibited high mechanical properties including flexural strength, fracture toughness, compressive strength and impact toughness. The intercoupling effect of layered structure and combination model of hard and soft played a key role in high impact resistance of the bionic layered composite, proving the feasibility and practicability of the bionic model of a *white clam* shell.

## 1. Introduction

Ceramic reinforced Al matrix composite is a kind of strong vitality material, which meets the demand of modern scientific development and engineering technology. The combination between high strength, hardness, elastic modulus and wear resistance of ceramic [1,2,3] and the low density, high ductility and toughness of Al matrix results in the comprehensive characteristics of light weight, high strength and high wear resistance [4,5,6]. Therefore, ceramic reinforced Al matrix composite is treated as one of the most promising candidate materials in aviation, aerospace, weapons, vehicles, ships and other fields of engineering components. However, the addition of common ceramic reinforcements such as TiC, TiB_2_, Al_2_O_3_, SiC and B_4_C [7,8,9,10] increases the specific strength and specific modulus, and decreases ductility and tenacity of Al matrix composite, reducing the impact resistance and enlarging the possibility of brittle rupture. The shortcomings of the ceramic reinforced Al matrix composite greatly limit its application in working conditions of high impact, stress and pressure [11,12]. The fabrication of ceramic reinforced Al matrix composite with the characteristics of high strength, hardness, ductility, light weight, high strength and impact resistance is crucial to improve the comprehensive performance of engineering components.

In the actual industrial production, many kinds of technologies have been adopted to fabricate ceramic reinforced Al matrix composite, such as powder metallurgy, stirring casting technique, melt infiltration process and flux assisted synthesis [13,14,15,16]. Peng and co-workers [17] fabricated TiC and Al_2_O_3_ reinforced Ni-Al matrix composite by hot pressed sintering. Compared with traditional ceramic/Al composite, the TiC-Al_2_O_3_/Ni-Al composite exhibited high strength and fracture toughness. Interface modification can improve the distribution of the ceramic and enhance bonding strength between reinforcement and matrix. In the scale of ceramic reinforcements, due to the high hardness, high elasticity modulus, good wettability, and relative stability with the Al matrix, TiC and TiB_2_ are expected to be the best reinforcements for Al matrix composites [7]. Moreover, TiC and TiB_2_ reinforced Al matrix composite can be fabricated from an Al-Ti-B_4_C system by self-propagating high temperature synthesis (SHS) directly. Shen and co-workers [18] analyzed the reaction mechanism and behavior of the Al-Ti-B_4_C system. The dissolution-diffusion-precipitation mechanism resulted in the perfect metallurgical bonding between TiC, TiB_2_ and Al matrix. Zhou and co-workers [19] prepared B_4_C/2024Al composite with a volume fraction of 45% by a pressure infiltration method. Under the impact of 7.62 mm penetrator, B_4_C/2024Al composite exhibited high impact resistance. Choi and Rhee [20] investigated the effect of Al content on morphology of TiC fabricated from the Al-Ti-C system. When Al content increased from 0 wt. % to 40 wt. %, TiC morphology changed from sintered shape to sphere shape. Particle size of TiC decreased from 15 to 0.4 µm. These technologies focus on matrix modification, interface modification, ceramic phase modification and improvement of the fabrication method. Though the bonding strength, wettability and mechanical properties have been improved, the rigidity enhancement, strength and impact resistance under high impact force is limited.

With the progress of science and human society, bionic research has changed the development of technologies and human civilization. Many kinds of biology such as shells, desert lizard, wood, bamboo, bone and tooth of animals and so on have light weight, high strength and impact resistance properties, providing important references for improving properties of ceramic/Al composite. After investigating the microstructure of *Crysomallon squamiferum*, Yao and co-workers [21] found that the characteristic of layered structure enhanced its properties of impact resistance, crack arrest and heat insulation. The structure characteristic of *Crysomallon squamiferum* has been treated as a bionic model for anti-impact material. Weaver and co-workers [22] studied the dactyl club of *Odontodactylus scyllarus* which owned high-velocity impact characteristics. The characteristics of a multiphase composite of oriented crystalline hydroxyapatite and amorphous calcium phosphate and carbonate and characteristics of a highly expanded helicoidal organization of the fibrillar chitinous organic matrix played an important role in the biological property of *Odontodactylus scyllarus*, which built a bionic model for the fabrication of armor. The biologies with light weight, high strength and high impact resistance provide a solution method for structure design and combination between light weight and high anti-impact of impact resistance material.

In our previous study [23], the microstructure and mechanical properties of *white clam* shell has been investigated. *White clam* shell with perfect mechanical and crack arrest properties consisted of a horny layer, prismatic layer and nacreous layer. Compared with other kinds of shells with good mechanical property, the *white clam* shell displayed relative light weight and high flexure and compression strength, which met the need of a bionic model for design of bionic layered composites.

Based on the principle of bionics, a *white clam* shell is chosen as the bionic model of this paper. TiC/TiB_2_-Al composite fabricated from Al-Ti-B_4_C system is chosen as main material of bionic layered material. Based on the light weight, high strength and impact resistance characteristics of the *white clam* shell, a kind of bionic layered impact resistance material of Al-Ti-B_4_C system is fabricated by corporation method of combustion synthesis and hot pressing sintering. This bionic layered material possesses the capabilities of light weight, high strength and impact resistance and provides a new idea, approach and method for the application of ceramic/Al composite in engineering.

## 2. Experimental Procedure

### 2.1. Material

The sample used for investigation of microstructure of *white clam* shell was purchased in the aquatic product market of Changchun, China. The soft tissues of *white clams* were removed by a scalpel carefully. Then the shells were rinsed by distilled water, and dried at room temperature for three days. Due to the low curvature and high mechanical properties, the sampling position was close to the wide marginal edge. The test sample for microstructure observation was prepared by breaking fragments from the *white clam* shells and observed scanning electron microscopy (SEM) (Model Evo18 Carl Zeiss, Oberkochen, Germany). More details about preparation of *white calm* shell samples can be found in our previous investigation [23].

The starting materials were purchased from commercial powders of aluminum (99.5% purity ~48 µm), titanium (99.5% purity ~25 µm) and boron carbide (99.9% purity ~13 µm), respectively. In this study, the molar ratio of Ti and B_4_C in Al-Ti-B_4_C system was 3:1. In order to realize bionic design and formability of bionic layered composite, the Al content of Al-Ti-B_4_C system varied from 30 to 50 wt. % of the total weight of the mixture. The reactant powders were mixed in a stainless steel container using stainless-steel balls at a low speed (~35 rpm) for 8 h to ensure homogeneity. According to the characteristics of *white clam* shell, a bionic model was built to guide the arrangement of Al-Ti-B_4_C layers with 30 wt. %, 40 wt. % and 50 wt. % Al contents. To meet the dimension demand of mechanical tests and practical fabrication feasibility, bionic layered composites with Φ 85 mm × 10 mm (30 g 40 wt. % Al-Ti-B_4_C, 10 g Al, 30 g 50 wt. % Al-Ti-B_4_C, 10 g Al and 30 g 30 wt. % Al-Ti-B_4_C) and Φ 85 mm × 6 mm (20 g 40 wt. % Al-Ti-B_4_C, 5 g Al, 20 g 50 wt. % Al-Ti-B_4_C, 5 g Al and 20 g 30 wt. % Al-Ti-B_4_C) were prepared. The 40 wt. % Al-Ti-B_4_C, Al, 50 wt. % Al-Ti-B_4_C, Al, 30 wt. % Al-Ti-B_4_C were laid into a graphite die and pressed into a plane surface successively. The arrangement mode of the layers of pure Al powder and the layers of Al-Ti-B_4_C powder was alternate. Al-Ti-B_4_C layers with different Al content were occupied the outer layer, middle layer and inner layer, respectively. Pure Al layers were arranged between arbitrary two Al-Ti-B_4_C layers, forming the layered pattern of precast block.

The combined precast block was pressed into cylindrical compact (about 85 mm in diameter) using a graphite die to obtain the density of 65% ± 2% theoretical density. Then the graphite die with compact was put into an intermediate frequency furnace under air atmosphere. The layered cylindrical compacts were heated to the ignition temperature of SHS reaction of Al-Ti-B_4_C systems. During the heating process, the temperature of graphite die was measured by an infrared temperature measuring sensor (Asmik, Hangzhou, China). The judge of occurrence of SHS in the ignition process was the sharp incensement of temperature sensor resulting from the heat released from reactions of Al-Ti-B_4_C systems. Then the graphite die was in progress of heat preservation for 5 min at ignition temperature of the infrared temperature measuring sensor. When the temperature decreased and was maintained at fixed point of Al for 5 min, the pressure of 6 t was applied on graphite die to increase the density of the composite. After 5 min of pressure preservation which followed by a cooling progress, the composite was prepared for metallographic and mechanical experiments. In order to exhibit changes in impact resistance property of bionic layered composite, the homogeneity Al-Ti-B_4_C composite was fabricated under the same preparation conditions.

### 2.2. Metallographic Experiment

Metallographic sample with dimension of 10 mm × 10 mm × 10 mm (Length × Width × Thickness) was prepared by a wire-electrode cutting machine to carry out metallography experiment. The samples were ground to get a smooth surface and etched by Keller’s reagent (1 vol. % HF + 1.5 vol. % HCl + 2.5 vol. % HNO_3_ + 95 vol. % H_2_O). After ultrasonically cleaning in alcohol for 10 min, the specimens were dried in hot air to obtain a clean surface.

### 2.3. Mechanical Practice

Flexural strength, fracture toughness, compressive strength and impact toughness are the key components of mechanical practices used to evaluate the impact resistance property. Therefore, the corresponding mechanical practices were conducted. Due to the ingredient diversity in axial direction of bionic layered composite, the loading direction of the mechanical tests was applied from the outer layer (40 wt. % Al-Ti-B_4_C) to inner layer (30 wt. % Al-Ti-B_4_C).

#### 2.3.1. Microhardness Test

In order to understand hardness variation of different layers of bionic layered composite, a microhardness testing machine (HVS-1000, Shanghai Jujing Precision Instrument Manufacturing Co., Ltd., Shanghai, China) was used to measure hardness by applying a load of 100 g for 5 s on the vertical section. Five measurements were made for each specimen to get the average hardness value.

#### 2.3.2. Flexural Strength Test

Three-point bending tests were used to obtain the flexure strength of bionic layered composite. The flexure strength was calculated by Equation (1).

(1) $$\sigma =\frac{3\text{PL}}{2{\text{bh}}^{2}}$$

where σ represented the flexure strength. P was the critical load during bending experiment process. L, b and h were the length of the support span, width and thickness of the samples, respectively.

Combine the actual situation of fabrication of bionic layered composite and standard size requirement of flexural strength sample, homogeneity Al-Ti-B_4_C composite and bionic composite specimen were cut into dimensions of 30 mm × 10 mm × 10 mm (Length × Width × Thickness) by the wire-electrode cutting machine. After grounding to get the smooth surface, a universal testing machine (Model DDL-100, Changchun, China) with the constant loading rate of 0.2 mm/min was employed to test flexure strength. The loading span was 24 mm. Average value of flexural strength was calculated from three individual measurements.

#### 2.3.3. Fracture Toughness Test

Ceramic reinforced metal matrix composites were sensitivity to crack. Fracture toughness can measure the crack arrest characteristic of the composite, especially for single edge notched beam method. Equation (2) was used to calculate fracture toughness. The selection of corresponding data was conducted in Reference [24].

(2) $$K_{I}=Y\frac{3\text{PL}}{{2\text{bh}}^{2}}\sqrt{a}$$

where K_I_ represented fracture toughness. Y was the geometry factor of the specimen. P was the critical load. L, b and h were the length of the support span, width and thickness of the samples, respectively. a was notch depth.

Fracture toughness was tested by a universal testing machine with a loading rate of 0.2 mm/min (Model DDL-100, Changchun, China). Samples with dimension of 30 mm × 10 mm × 6 mm (Length × Width × Thickness) was cut off by a wire-electrode cutting machine, and grounded to get the smooth surface. The depth and width of notch was 1.3 mm and 0.2 mm, respectively. The loading span for three-point bending tests was 24 mm. Under the condition of 0 ≤ a/h ≤ 0.6, value of Y can be calculated by the following equation [24]. Value of A_0_, A_1_, A_2_, A_3_ and A_4_ are shown in Table 1. Fracture toughness was obtained from the average value of three parallel tests.

(3) $$Y=A_{0}+A_{1}\frac{a}{h}+A_{2}{\left(\frac{a}{h}\right)}^{2}+A_{3}{\left(\frac{a}{h}\right)}^{3}+A_{4}{\left(\frac{a}{h}\right)}^{4}$$

#### 2.3.4. Compressive Strength Test

The anti-compression property of bionic layered composite and Al-Ti-B_4_C composite was valued according to compression strength. The following equation was used to calculate the compression strength.

(4) $$\sigma =\frac{P}{A}$$

where σ represented the compression strength. P was the critical load during compression experiment process. A was the cross sectional area. The bionic and homogeneity composites were cut into a circular column of Φ5 mm × 10 mm by the wire-electrode cutting machine, and grounded to get the smooth surface. Compression strength was tested by a universal testing machine (Model DDL-100, Changchun, China). The constant loading rate was 0.2 mm/min. The average value of the compression strength was got from three individual tests.

#### 2.3.5. Impact Toughness Test

Impact toughness is crucial to measure the impact resistance of bionic composite. Standard Charpy U-notch specimen was used for impact resistance experiment. The impact resistance sample with dimension of 50 mm × 10 mm × 10 mm (Length × Width × Thickness) was cut off by the wire-electrode cutting machine, and grounded to get the smooth surface. The impact toughness was calculated by the following equation.

(5) $$a=\frac{A}{\text{bh}}$$

where a represented impact toughness. A was ballistic work. b and h were width and thickness of specimen. A can be obtained from impact testing machine (Model RPK450, Changchun, China). The loading span for impact toughness tests was 40 mm. The average value of the compression strength was obtained from three individual tests.

The metallography and fracture morphology of bionic composite were examined using scanning electron microscopy (SEM) (Model Evo18 Carl Zeiss, Oberkochen, Germany). The phase component was identified using X-ray diffraction (XRD) (Model D/Max 2500PC, Rigaku, Tokyo, Japan).

## 3. Results and Discussion

### 3.1. Microstructure Characteristic of Bionic Model

**Figure 1:**
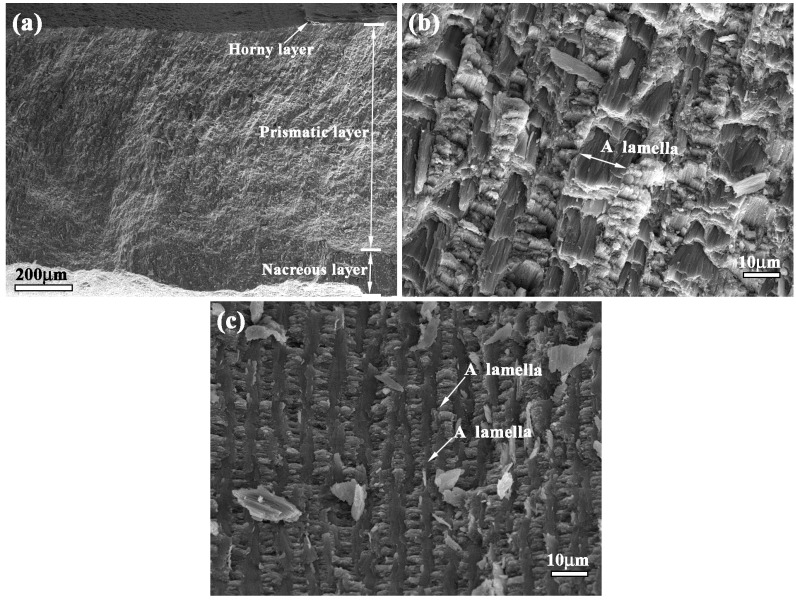
Microstructure of *white* clam shell (**a**) horny layer (**b**) prismatic layer and (**c**) nacreous layer.

Figure 1 shows the microstructure of a *white clam* shell. *White clam* shell can be divided into three layers: horny layer, prismatic layer and nacreous layer, as shown in Figure 1a–c, respectively. From Figure 1a, it can be found that the thickness of the prismatic layer occupies most of the whole thickness of the shell. The prismatic layer consists of various lamellae. The extension direction of a lamella crisscrosses the adjacent ones, exhibiting the typical characteristics of crossed-lamellar structure, as shown in Figure 1b. Compared with Figure 1a,b, the nacreous layer exhibits different surface morphology. The nacreous layer also consists of crossed lamellae, conforming to the characteristics of crossed-lamellar structure. Even though the extension direction of lamellae in the prismatic layer is different from that in nacreous layer, the microstructure of a *white clam* shell is characterized by crossed-lamellar structure.

In our previous study [23], the effect of microstructure, microhardness gradient on the mechanical properties of *white clam* shell was disclosed. The microhardness values of horny layer, prismatic layer and nacreous layer were 273 HV, 240 HV and 300 HV, respectively [23]. Different layers presented different hardness values, forming alternation combination model of hard and soft. The hardness value of outer layer was greater and less than that of middle layer and inner layer, respectively, constituting the typical characteristic of combination model of hard and soft. The coupling effect between layered microstructure properties and combination model of hard and soft played a key role in enhancing the mechanical properties of the *white clam* shell. Based on microstructure characteristics, *white clam* shell possessed light weight and high strength properties, which satisfied demands of a bionic model for designing a bionic layered composite.

### 3.2. Bionic Coupling Impact Resistance Model

**Figure 2:**
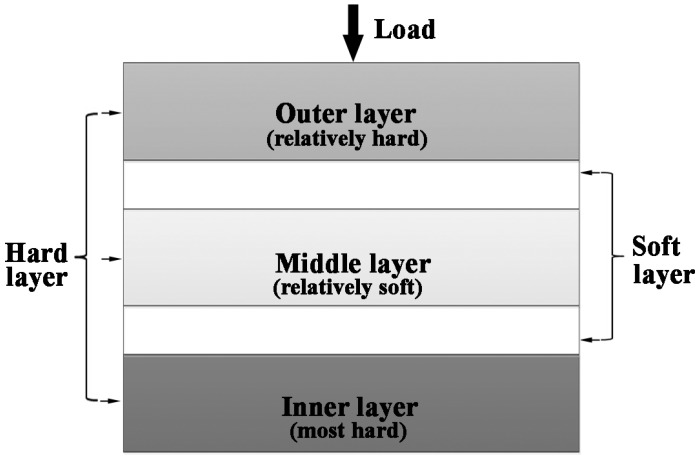
Diagrammatic sketch of bionic coupling impact resistance model.

According to the coupling effect between layered microstructure properties and the combination model of a hard and soft of *white clam* shell, a simplified diagrammatic sketch of a bionic coupling impact resistance model is built to guide the design of bionic layered composite, as shown in Figure 2.

The bionic coupling impact resistance model consists of three hard layers and two soft layers. Soft layers space the contiguous two hard layers, connecting hard layers. Hard layers own different intensities of colors, which represents different hardness values. The hardness value of the outer layer is greater and less than that of middle layer and inner layer, respectively, which is consistent with the hardness tendency of *white clam* shell. The load is applied from outer layer to inner layer, which is similar to the loading direction of the *white clam* shell. Intercoupling the model between the hard layer and soft layer is identical to the layered microstructure property and hardness tendency of *white clam* shell. Moreover, the bionic coupling impact resistance model ensures the feasibility of the actual processing, establishing the connection between bionic design and bionic fabrication.

### 3.3. Microstructure and Phase Identification

#### 3.3.1. Metallography and Microhardness

**Figure 3:**
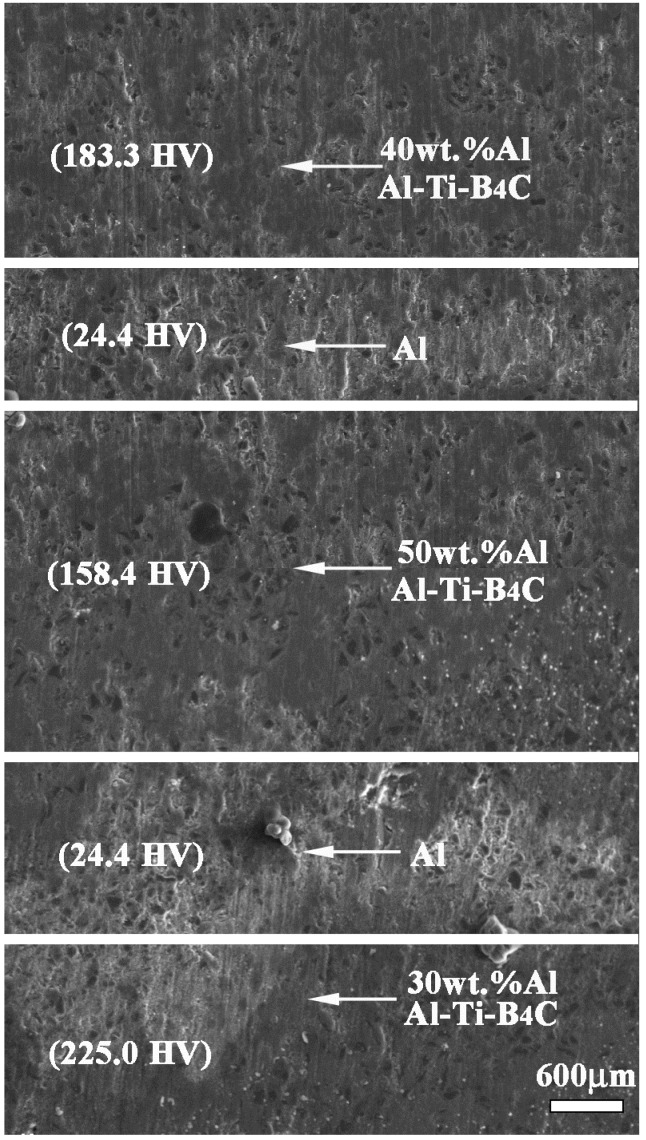
Metallography and microhardness of bionic layered composite.

Figure 3 shows the metallography and corresponding microhardness of bionic layered composite. TiC-TiB_2_ reinforced Al matrix composites fabricated from Al-Ti-B_4_C system with 30 wt. %, 40 wt. % and 50 wt. % Al content are treated as hard layers in Figure 2. Pure Al matrix is treated as soft layers. Variation of Al contents significantly affects hardness value of Al-Ti-B_4_C composite. Compared with Al matrix (24.4 HV), microhardness values of 30 wt. %, 40 wt. % and 50 wt. % Al-Ti-B_4_C composites are 225 HV, 183.3 HV and 158.4 HV, respectively. With the increase of Al content, hardness values of Al-Ti-B_4_C composites decrease clearly. The diversity of hardness of 30–50 wt. % Al-Ti-B_4_C composites can realize the combination model of hard and soft in Figure 2. According to the distribution of different layers, Al-Ti-B_4_C composites and Al matrix can be organized into a layered structure in Figure 2. Namely, bionic layered composite with the characteristics of layered microstructure property and combination model of hard and soft can be fabricated in accordance with a bionic coupling impact resistance model, as shown in Figure 3.

The obvious layered phenomenon of bionic layered impact resistance can be found in metallography of Figure 3. Different hard layers exhibit different color depths, which represents different hardness. Combined with hardness values of 30–50 wt. % Al-Ti-B_4_C system, Al-Ti-B_4_C composites with 40 wt. %, 50 wt. % and 30 wt. % Al contents are the outer layer, middle layer and inner layer, respectively. The white layers are the soft layers of Al matrix. Bionic layered composite has high compactness and metallurgical bonding. From the point view of metallography, bionic layered composite is fabricated successfully in accordance with the bionic coupling impact resistance model.

#### 3.3.2. Microstructure

**Figure 4:**
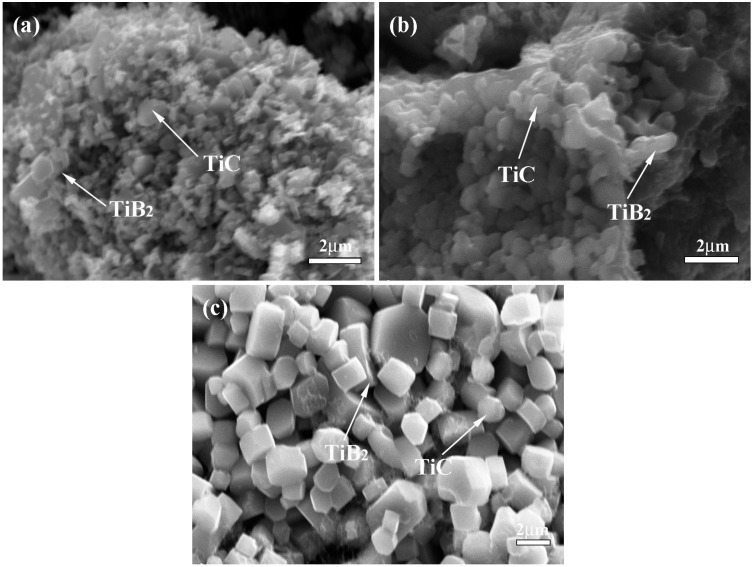
Fracture morphology of bionic layered composite (**a**) outer layer (**b**) middle layer (**c**) inner layer.

Figure 4 shows the fracture morphology of the outer layer, middle layer and inner layer in bionic layered composite. TiC ceramic particulate with near-spherical shape and TiB_2_ ceramic particulate with a nearly rectangular shape exhibit a uniform particle size in every particular Al-Ti-B_4_C layer. Variation of Al content affects ceramic particle size. With the increase of Al content, ceramic particle size decreases. This phenomenon can be explained in the following way briefly. The SHS reaction mechanism of Al-Ti-B_4_C is called the dissolution-diffusion-precipitation mechanism [7,18], resulting in the formation of TiC and TiB_2_. The growth of TiC and TiB_2_ is influenced by dwell time at high temperatures and maximum combustion temperature. The higher combustion temperature and longer dwell time promote the ceramic growth. The growth of TiC and TiB_2_ is an exponential function of the combustion temperature. The increase of Al content decreases the dwell time at high temperature and increases the liquid metal surrounding the ceramic particulates, which reduces the driving force for ceramic particulates growth and prevents sintering among ceramic particulates to form larger particulates. Therefore, with the increase of Al content, the size of the TiC and TiB_2_ particulates decreases. More details about the explanation for this phenomenon are given in previous studies [7,18,25]. Analysis of fracture morphology confirms that the TiC-TiB_2_ reinforced Al matrix composite is prepared successfully in every hard layer of bionic layered composite, which builds a substantial base with high mechanical property.

#### 3.3.3. Phase Identification

**Figure 5:**
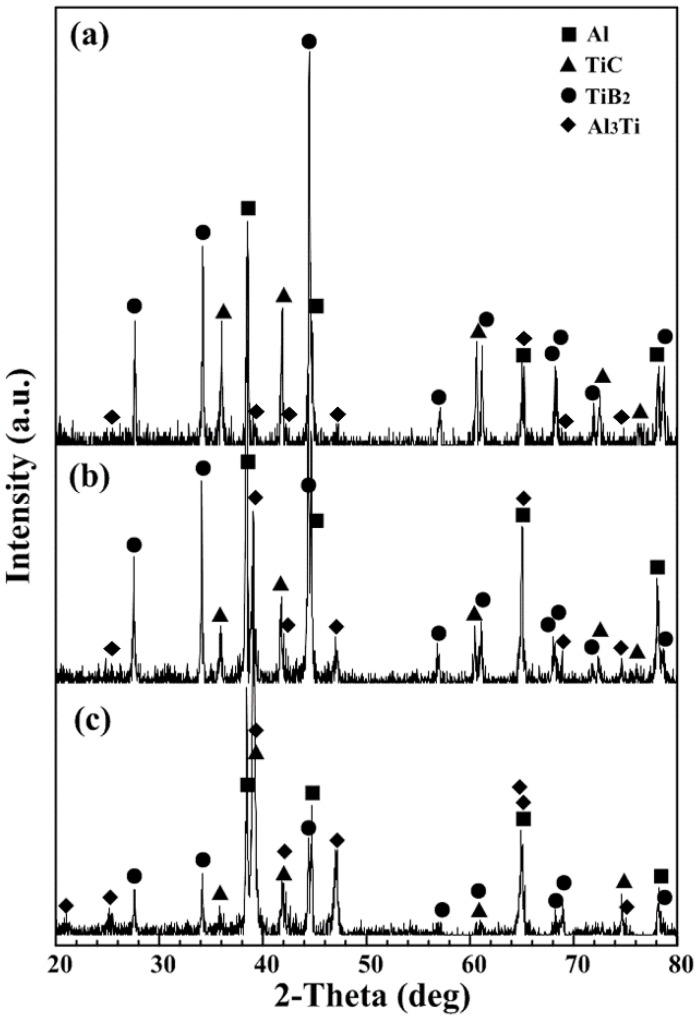
Phase identification of bionic layered composite (**a**) outer layer (**b**) middle layer (**c**) inner layer.

Phase identification of the outer layer, middle layer and inner layer in bionic layered composite are shown in Figure 5. As indicated, the outer layer (40 wt. % Al-Ti-B_4_C system), middle layer (50 wt. % Al-Ti-B_4_C system) and inner layer (30 wt. % Al-Ti-B_4_C system) of bionic layered composite consist of Al, TiC, TiB_2_ and Al_3_Ti, as shown in Figure 5a–c. The existence of Al_3_Ti enhances the metallurgical bonding between reinforcements and matrix. The reasons for the appearance of Al, TiC, TiB_2_ in bionic layered composite can be briefly explained as follows. SHS reaction of Al-Ti-B_4_C system began with the formation of Ti_x_Al_y_ compound via solid state diffusion. The C atom of B_4_C disassociated and diffused in Ti_x_Al_y_ liquid phase, resulting in the formation of Al-Ti-B-C quaternary liquid with low consistence of the B atom. Due to the interface between B_4_C and Al-Ti liquid, there existed a high amount of C, TiC formed firstly on the surface of B_4_C particulate. With the increase of temperature, C and B dissolved and diffused into the liquid phase, forming the Al-Ti-B-C quaternary liquid with high consistence of B atom. TiC and TiB_2_ precipitated from the quaternary liquid phase. Therefore, the product of Al-Ti-B_4_C system consisted of Al, TiC, TiB_2_. Moreover, details about the SHS reaction process can be found in previous studies [7,18]. Even though bionic layered composite was fabricated under air, no existence of oxides can be found in the XRD result.

The phase identification confirmed the role of main material of Al-Ti-B_4_C composite in bionic layered material. From the point view of phase identification, the bionic layered composite was fabricated successfully in accordance with the bionic coupling impact resistance model. The fabrication of the bionic layered impact resistance composite verified the practicability of the bionic coupling impact resistance model. The high bonding strength of every layer and steady phase component played an important role in mechanical property, especially impact resistance. Therefore, based on characteristics of microstructure and phase components, impact resistance and mechanical properties including flexural strength, fracture toughness, compressive strength and impact toughness of bionic layered impact resistance composite were investigated.

### 3.4. Mechanical Properties

#### 3.4.1. Flexural Strength

**Figure 6:**
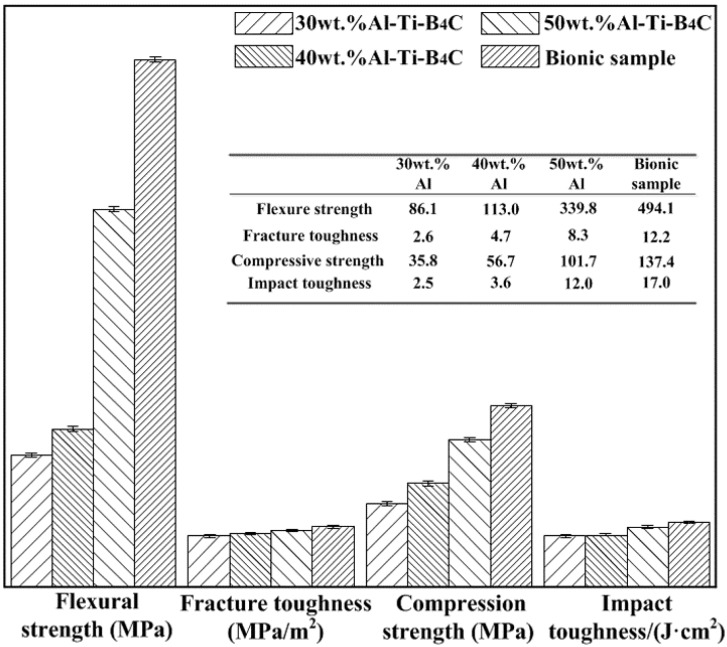
Flexural strength, fracture toughness, compression strength and impact toughness values of 30 wt. %–50 wt. % Al-Ti-B_4_C composite and bionic layered composite.

Flexural strength, fracture toughness, compression strength and impact toughness values of bionic layered composite are shown in Figure 6. Flexure strength values of Al-Ti-B_4_C composites with 30 wt. %, 40 wt. % and 50 wt. % Al contents are 86.1 MPa, 113.0 MPa and 339.8 MPa, respectively. With the increase of Al content, flexure strength of Al-Ti-B_4_C composites increases. The flexural strength value of bionic sample is 494.1 MPa, which is higher than that of 30–50 wt. % Al-Ti-B_4_C homogeneity materials.

**Figure 7:**
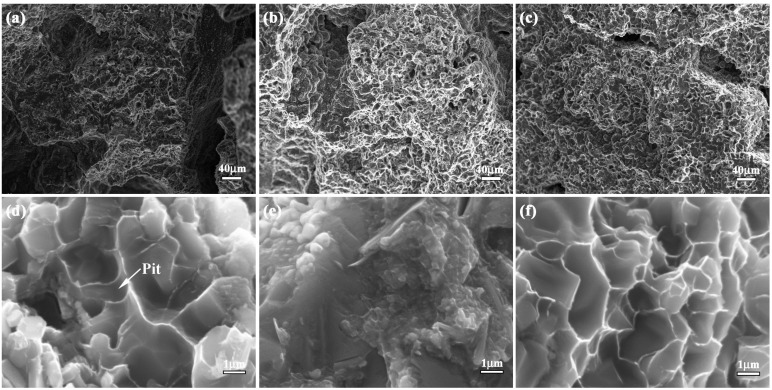
Flexural appearance of bionic layered composite (**a**) outer layer (**b**) middle layer (**c**) inner layer (**d**) magnified microstructure of (**a**); (**e**) magnified microstructure of (**b**); and (**f**) magnified microstructure of (**c**).

In order to understand the effect of layered microstructure and combination model of hard and soft layers on flexural strength of bionic impact resistance composite, the flexural appearance was studied. The corresponding results are shown in Figure 7a–f. The three layers of bionic sample exhibit different morphology characteristics. Several long and wide cracks appear on the outer layer. There is no existence of continuous and destructive cracks on middle and inner layer. In the magnified microstructure of three layers, a number of pits with different size exist on flexural appearance. When Al content increases from 30 to 50 wt. %, the size of the pits decreases.

The analysis of flexural appearance of bionic layered composite indicated that variation of Al content significantly affected flexural strength. Under the applied bend load, the outer layer of 40 wt. % Al-Ti-B_4_C composite endured the highest applied load, resulting in the existence of long and wide cracks. The addition of soft layer of Al absorbed some load and released damage of middle and inner layer. Therefore, no continuous and destructive cracks can be found in Figure 7b,c. The three layers of bionic composite defused the applied load by intergranular fracture, leading to the existence of a pit. With the increase of Al content, ceramic particle size decreased, which resulted in the decrease of the size of the pits in Figure 7. Combined with Figure 6, layered structure and combination model of hard and soft significantly enhance flexural strength of the bionic layered composite.

#### 3.4.2. Fracture Toughness

**Figure 8:**
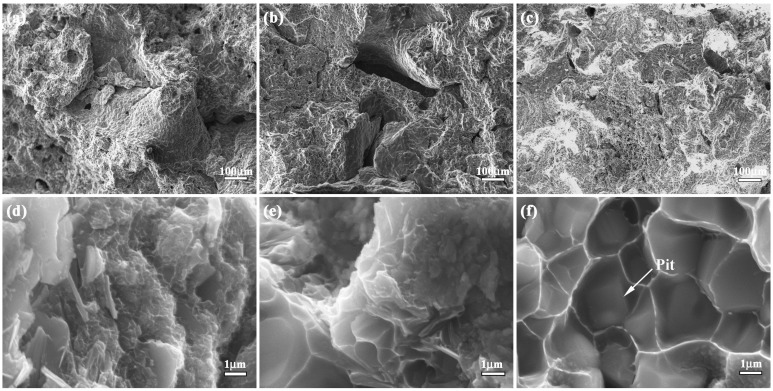
Fracture appearance of bionic layered composite (**a**) outer layer (**b**) middle layer (**c**) inner layer (**d**) magnified microstructure of (**a**); (**e**) magnified microstructure of (**b**); and (**f**) magnified microstructure of (**c**).

Fracture toughness is a key parameter to measure the crack arrest property of the material. Fracture toughness of bionic sample (12.2 MPa/m^2^) is almost 4.7, 2.6 and 1.5 times as great as Al-Ti-B_4_C composite with 30 wt. % (2.6 MPa/m^2^), 40 wt. % (4.7 MPa/m^2^) and 50 wt. % (8.3 MPa/m^2^) Al content, respectively, as shown in Figure 6. Identification of fracture appearance is beneficial to understand the crack arrest property of bionic layered composite. The corresponding result is shown in Figure 8a–f. Fracture appearance of three layers in bionic layered composite present irregular morphology and a number of small pits with different size and cracks.

In the initial stage of bend progress, the applied load led to the extension of crack in the position of the crack presented in the specimen. During the process of crack extension, most TiC and TiB_2_ crystalline grains in three layers were extracted, leading to intergranular fracture phenomenon and irregular morphology. When the cracks reached the soft layers, the toughness fracture of Al restricted crack extension and enhanced fracture toughness. Due to the intercoupling model between the hard layer and soft layer, the bionic layered composite exhibited the perfect crack arrest property and interlayer bonding.

#### 3.4.3. Compressive Strength

Compression strength values of 30–50 wt. % Al-Ti-B_4_C composites and bionic layered composite can be found in Figure 6. Compressive strength of bionic sample is 137.4 MPa, which is higher than that of Al-Ti-B_4_C composite. Bionic layered composite possesses the highest compressive strength, and exhibits perfect compression resistance. Variation of Al content influences the compressive strength of Al-Ti-B_4_C composites obviously. The compressive strength values of TiC-TiB_2_ reinforced Al matrix composites with 30 wt. % Al, 40 wt. % Al and 50 wt. % Al content are 35.8 MPa, 56.7 MPa and 101.7 MPa, respectively.

**Figure 9:**
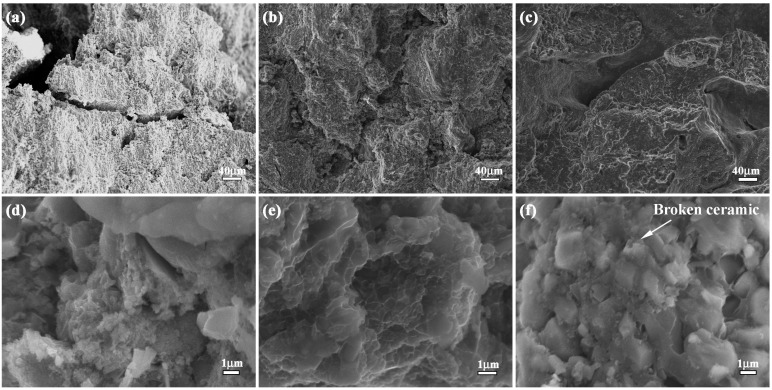
Compression appearance of bionic layered composite (**a**) outer layer (**b**) middle layer (**c**) inner layer (**d**) magnified microstructure of (**a**); (**e**) magnified microstructure of (**b**); and (**f**) magnified microstructure of (**c**).

Compression appearance and corresponding magnified microstructure of the three layers in bionic layered composite are shown in Figure 9a–f. Bionic composite presents an irregular compression appearance. A large number of pits resulted from intergranular fracture phenomenon of TiC and TiB_2_ particles exist on compression appearance. Several wide and serious cracks cross the outer layer and middle layer. In the magnified compression appearances, the broken ceramics, spalling fragments and pits exhibit different roles of hard and soft layers in bionic layered composite during the compression process.

During the compression progress, TiC and TiB_2_ particles were extracted and broken by the compression load, resulting in pits and cracks to defuse load. The existence of pits and cracks in Figure 9 indicated that the dominant fracture behavior of the three hard layers was the intergranular fracture. When cracks reached the soft layers, the high toughness of Al layers enhanced the compression strength of bionic sample and reset extension direction of cracks in the next layer. The layered structure and intercoupling model between the hard layer and soft layer led to a higher compression strength than that of traditional homogenous Al-Ti-B_4_C composite.

#### 3.4.4. Impact Toughness

**Figure 10:**
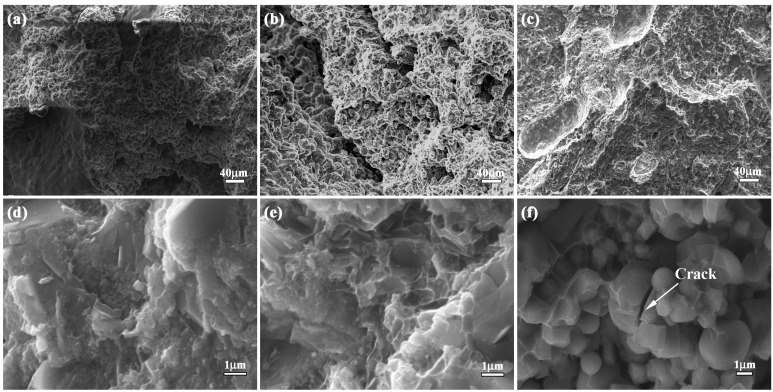
Impact appearance of bionic layered composite (**a**) outer layer (**b**) middle layer (**c**) inner layer (**d**) magnified microstructure of (**a**); (**e**) magnified microstructure of (**b**); and (**f**) magnified microstructure of (**c**).

Compared with traditional homogenous Al-Ti-B_4_C composite, bionic layered composite possessed high mechanical properties including flexural strength, fracture toughness and compressive strength, which builds its base of high impact resistance. Therefore, in order to investigate the impact resistance of bionic layered composite, the impact toughness and impact appearance were studied. The corresponding results are shown in Figure 6 and Figure 10, respectively.

Impact toughness of bionic layered composite is 17 J/cm^2^, which is higher than that of Al-Ti-B_4_C composites. Variation of Al content influences the impact toughness of Al-Ti-B_4_C composite with 30–50 wt. % Al content. Impact toughness values of homogenous 30 wt. %, 40 wt. % and 50 wt. % Al-Ti-B_4_C composites are 2.5 J/cm^2^, 3.6 J/cm^2^ and 12.0 J/cm^2^, respectively.

Figure 10a–f is the impact appearance and magnified microstructure of three layers in bionic layered composite. A large number of pits exist on the irregular impact appearance. It is worth noting that several dimples and cracked ceramic particles resulting from a certain degree of plastic deformation appear on the inner layer. In contrast to the inner layer, a crack is characteristic of the outer layer and middle layer of a bionic layered composite. Under the applied impact load, a bionic layered composite shows high interlayer bonding between the hard layer and soft layer. Analysis of impact appearance discloses the high impact resistance of the bionic layered composite.

After investigation of mechanical properties of bionic layered composite, it can be found that the bionic layered composite had relatively high flexural strength, fracture toughness, compression strength and impact toughness, which constructed a solid foundation for high impact resistance property. Under the applied impact load, due to mezzo mechanical properties, the outer layer of the bionic layered composite absorbed partial impact load in the form of an intergranular fracture. The soft layer of Al connected with the outer layer possessed high toughness, which restrained the appearance of the slabbing phenomenon. The middle layer of 50 wt. % Al-Ti-B_4_C composite possessed the lowest hardness and highest mechanical propertied among Al-Ti-B_4_C composites, which absorbed and defused most of the impact load. The soft layer connected with the middle layer absorbed impact load and reset the extension direction of cracks in next layer in the form of a ductile fracture. The highest hardness value of the inner layer of 30 wt. % Al-Ti-B_4_C composite played the role of fixing the position of the other layers and prevented delivery of the impact load. The intercoupling effect of the layered structure and combination model of hard and soft was the key point of the high impact resistance property of the bionic layered composite, which proved the feasibility and practicability of bionic design in Figure 2.

## 4. Conclusions

After summarizing the microstructure property of the *white clam* shell, a bionic coupling impact resistance model was established to guide the design of the bionic layered composite. The mechanical properties and impact resistance mechanism of the bionic layered composite fabricated by combination method of combustion synthesis and hot pressed sintering were investigated. The conclusions are described as follows:

(1) *White clam* shell with characteristic of crossed-lamellar structure can be divided into three layers: horny layer, prismatic layer and nacreous layer. The coupling effect between layered microstructure and combination model of hard and soft layers played the key role in enhancing mechanical properties of the *white clam* shell, which satisfied demands of a bionic model for design of bionic layered composite.

(2) A simplified bionic coupling impact resistance model was built to guide the design of the bionic layered composite. The intercoupling model between the hard layer and soft layer was identical to the layered microstructure property and hardness tendency of a *white clam* shell.

(3) Combined with hardness values of 30–50 wt. % Al-Ti-B_4_C system, Al-Ti-B_4_C composites with 40 wt. %, 50 wt. % and 30 wt. % Al contents were the outer layer, middle layer and inner layer, respectively. The soft layers were Al matrix. As the role of the main material in bionic layered material, fracture morphology and phase identification confirmed the successful fabrication of TiC-TiB_2_ reinforced Al matrix composite.

(4) Compared with traditional homogenous Al-Ti-B_4_C composite, high mechanical properties including flexural strength, fracture toughness and compressive strength formed the base of the high impact resistance of bionic layered composite. The intercoupling effect of layered structure and combination model of hard and soft layers was the key point of the high impact resistance property of the bionic layered composite, which proved to have the feasibility and practicability of bionic design contained in a *white clam* shell.

## Figures and Tables

**Table 1:** Value of A_0_, A_1_, A_2_, A_3_ and A_4_ [24].

Loading Mode	A_0_	A_1_	A_2_	A_3_	A_4_
Three-point bending (L/h = 4)	1.93	−3.07	14.53	−25.11	25.80
